# Short-term fasting before living kidney donation has an immune-modulatory effect

**DOI:** 10.3389/fimmu.2025.1488324

**Published:** 2025-02-20

**Authors:** Christiaan A. J. Oudmaijer, Daphne S. J. Komninos, Rutger A. Ozinga, Kimberly Smit, Nina E. M. Rozendaal, Jan H. J. Hoeijmakers, Wilbert P. Vermeij, Joachim G. J. V. Aerts, Jan N. M. IJzermans, Marcella Willemsen

**Affiliations:** ^1^ Erasmus MC Transplant Institute, Department of Surgery, Erasmus University Medical Center, Rotterdam, Netherlands; ^2^ Princess Máxima Center for Pediatric Oncology, Utrecht, Netherlands; ^3^ Oncode Institute, Utrecht, Netherlands; ^4^ Department of Pulmonary Medicine, Erasmus University Medical Center, Rotterdam, Netherlands; ^5^ Erasmus MC Cancer Institute, Department of Molecular Genetics, Erasmus University Medical Center, Rotterdam, Netherlands; ^6^ Institute for Genome Stability in Ageing and Disease, Medical Faculty, University of Cologne, Germany, and Cologne Excellence Cluster for Cellular Stress Responses in Aging-Associated Diseases (CECAD), Centre for Molecular Medicine Cologne (CMMC), University of Cologne, Cologne, Germany

**Keywords:** Short-Term Fasting, acute inflammatory response, immune response, cell population analysis, caloric restriction

## Abstract

**Background:**

Short-Term Fasting (STF) is an intervention reducing the intake of calories, without causing undernutrition or micronutrient-related malnutrition. It aims to systemically improve resilience against acute stress. Several (pre-)clinical studies have suggested protective effects of STF, marking the systemic effects STF can induce in respect to surgery and ischemia-reperfusion injury. In addition, STF also affects the number of circulating immune cells. We aim to determine the effect of STF on the abundance and phenotype of different immune cell populations.

**Methods:**

Thirty participants were randomly selected from the FAST clinical trial, including living kidney donors, randomized to an STF-diet or control arm. In an observational cohort sub-study we prospectively included 30 patients who donated blood samples repeatedly during study runtime. Using flow cytometry analyses, immune cell phenotyping was performed on peripheral blood mononuclear cells. Three panels were designed to investigate the presence and activation status of peripheral T cells, B cells, dendritic cells (DCs) and myeloid cells.

**Results:**

Eight participants were excluded due to sample constraints. Baseline characteristics showed no significant differences, except for fasting duration. Weight changes were minimal and non-significant across different time intervals, with slight trends toward long-term weight loss pre-surgery. Glucose, insulin, and β-hydroxybutyrate levels differed significantly between groups, reflecting adherence to the fasting diet. Flow cytometry and RNA sequencing analysis revealed no baseline differences between groups, with high variability within each group. STF changes the levels and phenotype of immune cells, reducing the abundance and activation of T cells, including regulatory T cells, increased presence of (naïve) B cells, and elevation of type 1 conventional DCs (cDC1s). In addition, a decrease in central memory T cells was observed.

**Discussion:**

In this study, we observed significant changes due to fasting in B cells, T cells, and DCs, specifically toward less specialized lymphocytes, suggesting an arrest in B and T cell development. Further research should focus on the clinical implications of changes in immune cells and significance of these observed immunological changes.

**Conclusion:**

STF results in reduced numbers and activation status of T cells and Tregs, increased presence of (naïve) B cells, and elevation of cDC1s.

## Introduction

Short-Term Fasting (STF) and Caloric Restriction (CR) are both interventions reducing the intake of calories without causing undernutrition or micronutrient-related malnutrition. Both aim to systemically improve resilience against acute stress (1–7), with fasting focused on a short exposure, and CR on the long term. CR lowers the risk of age-associated diseases, boosts health span, and extends lifespan across many organisms (1–6, 8–14). CR and STF have been an objective of mostly preclinical and animal studies, where it has shown preventive effects on genomic stress, ischemia reperfusion injury, acute stress conditions and ageing (5, 9, 12, 14–18). In a clinical setting, STF is mostly applied prior or in addition to treatment as nutritional preconditioning and can be performed in different regimens (19). STF induces a *Survival Response* after a day or two but requires a significant and stringent reduction of caloric intake. This *Survival Response* protects against different types of stress, e.g. genotoxic stress caused by oxidative DNA damage (5, 9–14, 18). Interest in STF has increased recently, as it has been proven feasible and safe in human trials (9, 18–26), and holds potential for alleviating the burden of treatment (19, 27, 28) and possibly enhancing it (19–21). Potential short- and long-term health benefits of reducing caloric intake have only been partly translated to and investigated in humans, but is a topic heavily investigated (19–22).

Mechanistically, STF suppresses the somato-, lacto-, and thyrotropic hormonal axes, causing a temporary attenuation of growth, while protective antioxidant defenses, stress resistance and maintenance- and resilience mechanisms are enhanced (5, 9, 10, 12, 14, 18, 29–34). Treatments damaging DNA, such as chemotherapy, lead to acute genotoxicity and accelerated cell death, thereby causing functional decline and aging in local and systemic areas. This damage might be alleviated by CR and/or STF. Additionally, STF may have benefits in other aspects of medical treatment, such as improving post-surgery recovery. Surgical procedures involving temporary lack of oxygen and nutrients followed by reperfusion, such as in organ transplantation, generate acute tissue damage, increased cell death and inflammation (35). Several pre-clinical studies have demonstrated the protective effects of STF in their respective models; marking the systemic effects CR can induce in respect to surgery and ischemia-reperfusion injury (IRI) (9, 18, 19, 23–26, 36–38). Surgical procedures can induce local and systemic effects, with secondary cell death and inflammation (35). Therefore, STF could entail a method of mitigating the effects of acute surgery-induced stress. Recently, a large prospective randomized controlled trial was initiated, investigating the benefit of STF before living kidney donation (39). Living kidney donors are an excellent group to further investigate the effect of STF, as the surgery is scheduled electively, and donors are screened extensively before living donation. As the topic of duration and stringency of STF has already been extensively investigated, this trial focuses on the clinical benefit gained, for both the donor and recipient.

Aside from the aforementioned responses on cell damage and inflammation, STF and CR may also directly affect the number of circulating immune cells (7, 40–42). The abundance and phenotype of peripheral immune cells serves as an indicator of the body’s immune response (42). Postoperative recovery can be significantly influenced by the immune cell count; elevated levels of e.g. neutrophils immediately after surgery indicate the extent of the acute inflammatory response (43). Conversely, a decrease in lymphocyte count could signify immunosuppression, potentially impacting the body’s ability to combat infections during postoperative recovery (7, 40–42). Monitoring the immune cell populations over time may offer insights into the body’s response to surgical stress. STF seems to affect the intravascular presence and distribution of T and B cells and it alters the presence, metabolic functions and inflammatory activity of monocytes (40–42, 44). The primary objective of this study is to determine the effect of STF on the circulatory abundance and phenotype of different immune cell populations.

## Methods

### Population

Thirty participants were randomly prospectively selected from the FAST clinical trial (39), an active trial at the Erasmus MC Transplant Institute and University Medical Center Groningen (UMCG), registered in the Netherlands Trial Register (45). Both hospitals are tertiary academic centers with extensive experience with living kidney donation and transplantation (46). The participants for this sub-study were included at the Erasmus MC site. Eligible kidney donors were subjected to the study procedures, including randomization to STF or the control group, while the recipients were included for the collection of post-transplant clinical data. Castor EDC (Amsterdam, the Netherlands) was used in accordance with laws and regulations, to centrally randomize study subjects. Computerized stratified block randomization was performed to determine allocation to a treatment group; stratification was employed for subject sex and center. Trial participants and care providers could not be blinded to the result of randomization, but statistical analysis was performed blindly. Patients who consented to participating in the FAST-Study were prospectively included in this sub-study if they checked the non-obligatory checkbox on the informed consent file, stating that they consented to the sub-study. At the moment of inclusion into this sub-study, the result of the randomization was not yet known. We prospectively included thirty patients in our observational cohort study, aiming to include 15 patients from the control group and 15 patients from the intervention group. In this sub-study we acquired blood samples repeatedly during study runtime, in addition to the standard of care and study procedures conducted due to general study participation (39).

### Flow cytometry

**Figure 1:**
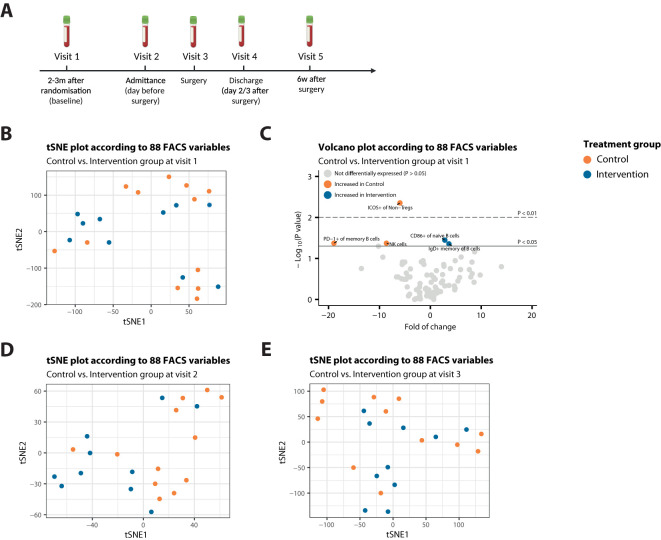
Patients from both treatment arms are highly comparable at start of treatment. **(A)** Schematic methodologic overview of the clinical procedures. The intervention group was instructed to follow a STF-diet, starting 60 hours before surgery. Participants drank ad libitum water, tea or coffee to maintain fluid balance, and to maintain electrolyte balance, they were allowed a max of 4 bouillon soups a day. After surgery, they resumed regular intake. **(B)** t-SNE plot that illustrates no sample segregation based on treatment among 88 FACS variables at Visit 1. **(C)** Volcano plot depicting the fold of change of 88 FACS variables between the two treatment groups at Visit 1. The x-axis represents the log2 fold change, whereas the y-axis represents -log10 P values. Variables on the right (positive) are higher in the intervention group, and those on the left (negative) are higher in the control group. **(D, E)** t-SNE plot that illustrates no sample segregation based on treatment among 88 FACS variables at Visit 2 **(D)** or Visit 3 **(E)**.

To investigate the immunological effects of STF, we conducted immune cell phenotyping using flow cytometry analyses on peripheral blood samples. Blood samples were collected at set time points: 2-3 months after randomization *(visit 1, baseline)*, on the day of admittance *(visit 2, the day before surgery)*, at the end of the surgical procedure *(visit 3)*, at the day of discharge from the hospital *(visit 4, day 2 or 3 after surgery)* and at follow-up 6 weeks after surgery *(visit 5)* (**Figure 1A**). All samples were drawn according to local protocol, sampled by trained medical personnel via venous puncture in three heparin tubes. From these samples, peripheral blood mononuclear cells (PBMCs) were isolated. PMBCs were purified from peripheral blood by Ficoll density gradient centrifugation (47) and cryopreserved until use.

Three panels were designed to investigate the presence and activation status of peripheral T cells, B cells, dendritic cells (DCs) and myeloid cells by flow cytometry (**Supplementary Table S1**). In short, PBMCs were stained for extracellular markers at 4°C for 30 minutes. Thereafter, cells were stained with Fixable Viability Dye (eBioscience) at 4°C for 15 minutes. After fixating at 4°C for 30 minutes using the FoxP3 Transcription Factor Staining Buffer Set (eBioscience), PBMCs were stained for intracellular markers at 4°C for 1 hour. Data was acquired on the FACSympony A5 using BD FACSDiva Software and analyzed by FlowJo™ Software (all BD Biosciences). The gating strategy for individual cell subsets is shown in **Supplementary Figure S1**. All data that followed from our FlowJo analysis are shown in **Supplementary Table S2**.

### Statistical analysis on immune cells and blood parameters

Adherence to the assigned randomization in our population was based on intake during admittance, self-reported adherence at admittance, by change in body weight during the fasting period and by blood samples drawn on the day of surgery. Relevant clinical data were collected from the electronic health record. At a set time point at admittance, after having followed the fasting diet for 2/3 of its intended duration, laboratory measurements such as fasting glucose (mmol/l), fasting insulin (pmol/l) and β-hydroxybutyrate (BHB)(mmol/l) were determined in order to quantify the metabolic effect of the diet. Statistical analysis was performed using R version 4.0.3 or newer. A two-sided significance level of 0.05 was used for all primary and secondary analyses, unless otherwise stated. Statistical tests (t-test, Chi-square, and Wald-test) were performed where applicable, depending on the type of variable. In addition, FACS data underwent t-distributed stochastic neighbor embedding (t-SNE) dimensionality reduction analysis, employing optimized hyperparameters (“perplexity” 1/4 18, “max_iter” 1/4 5,000, and “theta” 1/4 0). This facilitated the exploration of the data in a two-dimensional space, aiming to identify potential clustering driven by the treatment groups at different time points.

### RNA isolation and analysis

For transcriptome analysis, blood samples from both fasted and control patients were utilized (39). Sampling was done at the approximate same moment during the day of admittance to minimize variation due to differences in the circadian clock. Samples were snap frozen and RNA was isolated using the Monarch Total RNA Miniprep Kit (New England Biolabs; T2010) according to manufacturer’s protocol with on column DNase treatment. The RNA was further cleaned and concentrated using the RNA Clean & Concentrator-5 kit (Zymo Research; R1013). RNA quality and quantity were assessed using the NanoDrop One (Thermo Fisher Scientific). RNAseq library was constructed using KAPA Robo erase Hyperprep (Roche; 08098140702), with subsequent quantification by the Qubit (Thermo Fisher Scientific) and TapeStation system (Agilent) and finally total RNA was sequenced in house on the Illumina NovaSeq6000 system (Illumina).

Analysis of RNAseq raw data files was performed on our in house-generated data analysis pipeline (48). The reads were normalized using TMM normalization, followed by quantification of log2 fold changes and false discovery rates using EdgeR (version 3.40.2) and gene set enrichment analysis [GSEA, MSigDB, version 2023.2 (49, 50)] was performed using the GSEA function from the “cluster profiler” package [version 4.12.6 (51, 52)] to check the effects of fasting on specific processes. For GSEA, genes were ranked using the calculation -log10(pvalue)*log2(Fold change).

## Results

### Population

Inclusion for this sub-study started in November 2021 and was completed in November 2022. No intended FAST-Study participant objected to the acquirement of additional blood samples, with all 30 participants included consecutively. Eight participants were excluded from this analysis due to sample constraints. For six participants, the sample at admittance or at discharge was missing, due to logistical limitations, such as rescheduling the surgery and/or non-retrieval of the samples during regular rounds at the ward. For two participants, surgery for living kidney donation was not performed due to changes in the condition of the recipient. **Table 1** summarizes the clinical characteristics of the participants of which all five blood collections were available, and which were subsequently analyzed by flow cytometry. The control group consists of 13 individuals and the intervention group of 9 participants.

**Table 1:** Clinical characteristics of the patient cohort.

	Control (N=13)	Intervention (N=9)	Total (N=22)
Sex			
Female	6 (46.2%)	5 (55.6%)	11 (50.0%)
Male	7 (53.8%)	4 (44.4%)	11 (50.0%)
Smoking			
Never	6 (46.2%)	5 (55.6%)	11 (50.0%)
In tde Past	5 (38.5%)	3 (33.3%)	8 (36.4%)
Active	2 (15.4%)	1 (11.1%)	3 (13.6%)
Adherent			
Fully Adherent	13 (100.0%)	9 (100.0%)	22 (100.0%)
Non-Adherent	0 (0.0%)	0 (0.0%)	0 (0.0%)
Age			
Mean (SD)	49.7 (12.7)	47.2 (11.3)	48.7 (11.9)
Range	25 - 68	23 - 62	23 - 68
BMI			
Mean (SD)	27.6 (4.3)	26.2 (3.7)	27.0 (4.1)
Range	21.6 - 36.9	21.5 - 32.6	21.5 - 36.9
Fasting Duration 1 *(Hours)^a^*			
Mean (SD)	0 (0)	42.4 (0.7)	17.4 (21.4)
Range	0 - 0	41.0 – 43.0	0 – 43.0
Fasting Duration 2 *(Hours)^b^*			
Mean (SD)	12.2 (2.3)	61.0 (0.8)	32.1 (24.6)
Range	9 - 15.5	60.1 - 62.6	9 - 62.6

Clinical characteristics of the participants of which all five blood collections were available,

and which were subsequently analysed by flow cytometry.

^a^ Fasting Duration 1 corresponds with the moment of admittance/visit 2.

^b^ Fasting Duration 2 corresponds with the moment of surgery/visit 3.

SD, standard deviation; BMI, body mass index.

### Baseline characteristics

Analysis indicated no significant differences between the control and intervention group with regards to the clinical characteristics. There were an equal number of females (50.0%) and males (50.0%), evenly split between the control and intervention groups. Half of the participants were never-smokers, while 36.4% had a history of smoking and 13.6% were active smokers, with a similar distribution observed in both groups. Confirmation of diet adherence at admission revealed full compliance among all 22 participants. The average age was 49.7 years (SD=12.7) for the control group and 47.2 years (SD=11.3) for the intervention group, with an age range spanning from 23 to 68 years in both cohorts. Finally, the control and intervention group were also similar with regards to BMI. Examining the fasting duration at specific blood sampling points, the intervention group had a mean fasting duration of 42.4 hours at visit two/admittance, while the control group had adhered their regular diet routine. During surgery, both groups experienced the maximum fasting duration: the control group, following regular pre-surgery instructions, had fasted for an average of 12.2 hours, whereas the intervention group had fasted for an average of 61.0 hours.

### Change in weight

No significant weight difference was found between specific time intervals and there was minimal weight change overall. Specifically, analyses regarding weight showed no significant changes between moment of inclusion and 3 days before surgery (p = 0.245), inclusion and admittance (p = 0.325), and 3 days before surgery and admittance (p = 0.701), as seen in **Supplementary Figure S2A**. In addition to this, only subtle non-significant alterations in weight were observed for intervention and control groups. Despite a trend showing weight loss pre-surgery in both trial arms, it did not reach statistical significance. Weight variations occurred mostly during the waiting period, with variable and minor changes before surgery (**Supplementary Figures S2B, C**). Finally, the differences between study arms with regards to glucose, insulin, and β-hydroxybutyrate show notably lower glucose and insulin levels and higher β-hydroxybutyrate in the intervention group [p = 0.049, p = 0.001, and p = 0.001, respectively (**Supplementary Figures S2D–F**)], aligning with adherence to the fasting regimen.

### Clinical outcomes

Post-operative recovery of included living kidney donors was comparable between the two study-arms. Postoperative admission time was 3 days in the control arm (range 2-5), and 2.9 in the intervention arm (range 2-3). Postoperative complications arose in just one participant from the control arm and consisted of a superficial wound infection.

### STF-induced changes in peripheral immune cells

To exclude baseline differences between the intervention and control group, we performed t-distributed stochastic neighbor embedding (t-SNE) dimensionality reduction analyses. Exploration of flow cytometry data using t-SNE analyses revealed no segregation of baseline (Visit 1) blood samples, when stratified for study treatment (**Figure 1B**). Correspondingly, only 5 variables were significantly differentially expressed (DE) between the treatment groups at baseline (P<0.05) (**Figure 1C**), indicating that patients from both treatment arms were highly comparable at start of treatment, as also represented by the clinical characteristics shown in **Table 1**.

In line with no apparent segregation of patient samples at baseline, t-SNE analysis revealed no distinct clusters based on study treatment emerging after a mean fasting duration of 42.4 hours for the intervention group (**Figure 1D**), nor after the maximum fasting duration, (**Figure 1E**), indicating high inter-patient variability in every treatment group.

**Figure 2:**
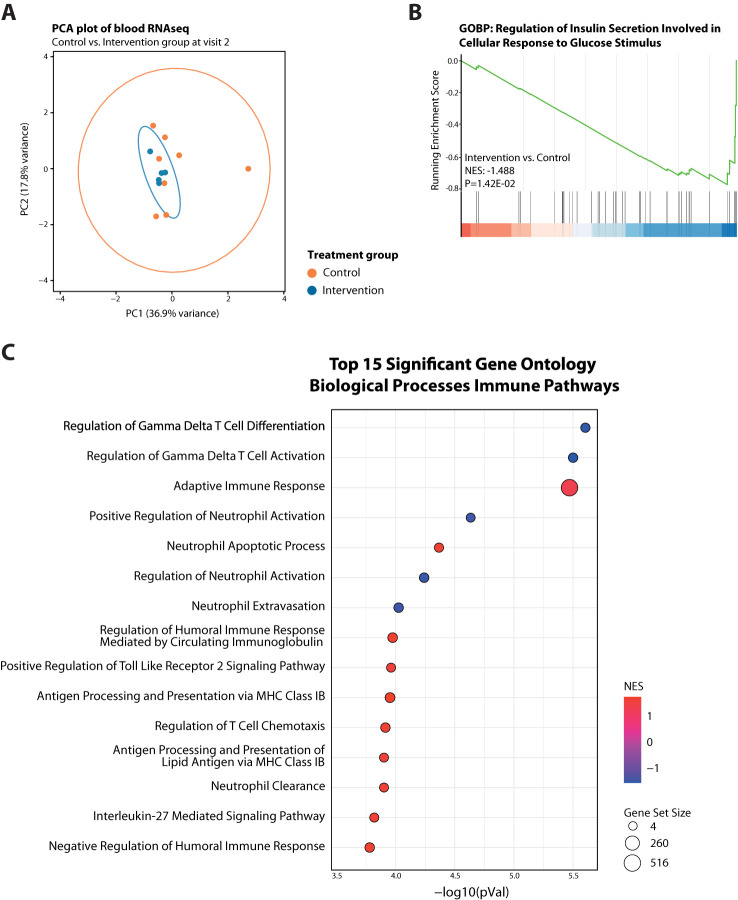
Transcriptome analysis of blood cells from fasted versus control participants at Visit 2. **(A)** PCA plot showing no separation between the two treatment arms at Visit 2 but high variability among controls. **(B)** GeneSet Enrichment Analysis (GSEA) plots of Gene Ontology Biological Process (GO-BP) Regulation of Insulin Secretion Involved in Cellular Response to Glucose Stimulus. **(C)** Bubble plot summary of GSEA top 15 significant GO-BP Immune Pathways. Red dots have a positive normalized enrichment score (NES), meaning upregulated, and blue dots negative NES, thus downregulated. Size of dots indicates size of corresponding gene set.

Next, we assessed a total RNA sequencing profile generated from total blood of 13 selected patients (8 controls and 5 fasted), isolated from the moment of admittance (Visit 2). Principal component analysis also showed no clear separation based on study treatment (**Figure 2A**), and was suggestive of high inter-patient variation for the control participants. Gene-set enrichment analysis (GSEA), considering all gene changes with a high sensitivity and low bias (48), revealed a suppression of insulin secretion involved in cellular response to glucose in the STF-intervention group compared to controls (**Figure 2B**), in line with their high adherence to the dietary regimens. Additionally, the intervention group revealed a clear change in immune related pathways and cell types, including suppressed gamma delta T cell differentiation and activation, neutrophil activation, and platelet activation signalling along with an enhanced adaptive immune response, antigen processing and regulation of T cell chemotaxis (**Figure 2C**; **Supplementary Figure S3**).

**Figure 3:**
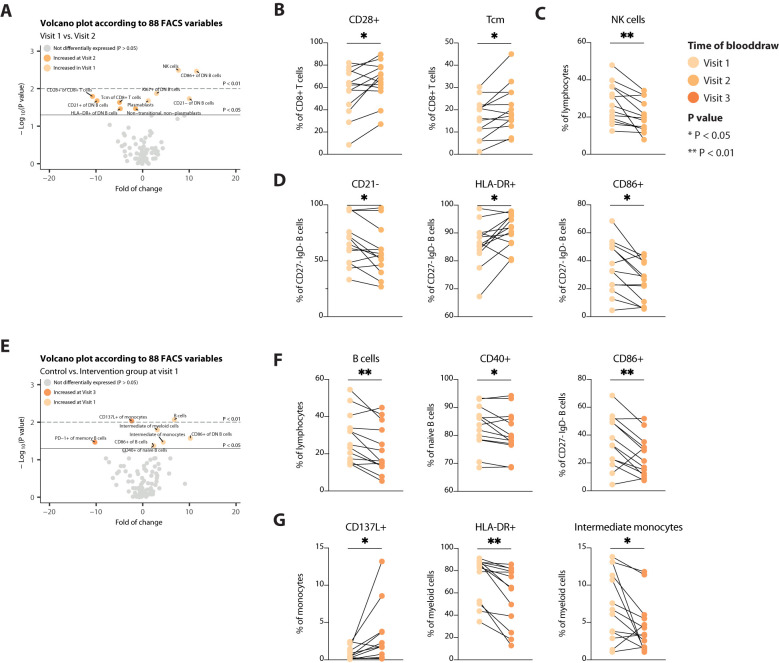
Assessment of alterations in immune cell populations over time in the control group. **(A)** Volcano plot depicting the fold of change of 88 FACS variables between the Visit 1 and Visit 2 in the control arm. The x-axis represents the log2 fold change, whereas the y-axis represents -log10 P values. Variables on the right (positive) are increased at Visit 1, and those on the left (negative) are increased at Visit 2. **(B-D)** Line plots showing alterations in T cells **(B)**, NK cells **(C)** and B cells **(D)**. **(E)** Volcano plot depicting the fold of change of 88 FACS variables between the Visit 1 and Visit 3 in the control arm. The x-axis represents the log2 fold change, whereas the y-axis represents -log10 P values. Variables on the right (positive) are increased at Visit 1, and those on the left (negative) are increased at Visit 3. **(F, G)** Line plots showing alterations in B cells **(F)**, and myeloid cells **(G)**. Tcm, central memory T cells. * = p < 0.05, ** = p <0.01.

Due to the high inter-patient variation, we next assessed alterations in immune cell populations over time within each patient. In the control arm of the trial, not adhering to a STF-Diet, minimal variables were significantly differentially expressed. Five were increased at day of admittance and five were decreased (**Figure 3A**). These involved expansion of CD8+ central memory T (Tcm) cells and increased expression of CD28+ on CD8+ T cells, indicating slight T-cell activation (**Figure 3B**). Tcm cells are long-lived antigen-experienced cells that provide enhanced protective response upon reinfection and recirculate between lymphoid organs and the blood. On the other hand, NK cells were decreased at day of admittance (**Figure 3C**). Concomitantly, patients in the control group showed phenotypic/cell surface changes in CD27- IgD- B cells (**Figure 3D**). These double negative (DN) B cells have been linked to autoimmunity, infectious diseases and cancer being either hyperresponsive, autoreactive or exhausted. DN B cells are diversely characterized, commonly also by lack of expression of CD21. We found that the frequency of CD21- cells within DN B cells was decreased at day of admittance (**Figure 3D**), suggesting less dysfunctional B cells, even though not accompanied by a decrease in DN B cells. Patients in the control arm did not show a definite profile with regard to more or less activation of DN B cells over time (**Figure 3D**), indicating the exact role of the cells remain inconclusive. During surgery, when the control group had a mean fasting time of 12.2 hours, none of these variables were still DE, except for the expression of CD86 on CD27- IgD- B cells (**Figures 3E-G**), suggesting less activation of DN B cells. The other DE variables mainly involved B cells and myeloid cells (**Figures 3F, G**) and were indicative of decreased B cell abundance and activation (the latter measured by CD40 and CD86 expression).

**Figure 4:**
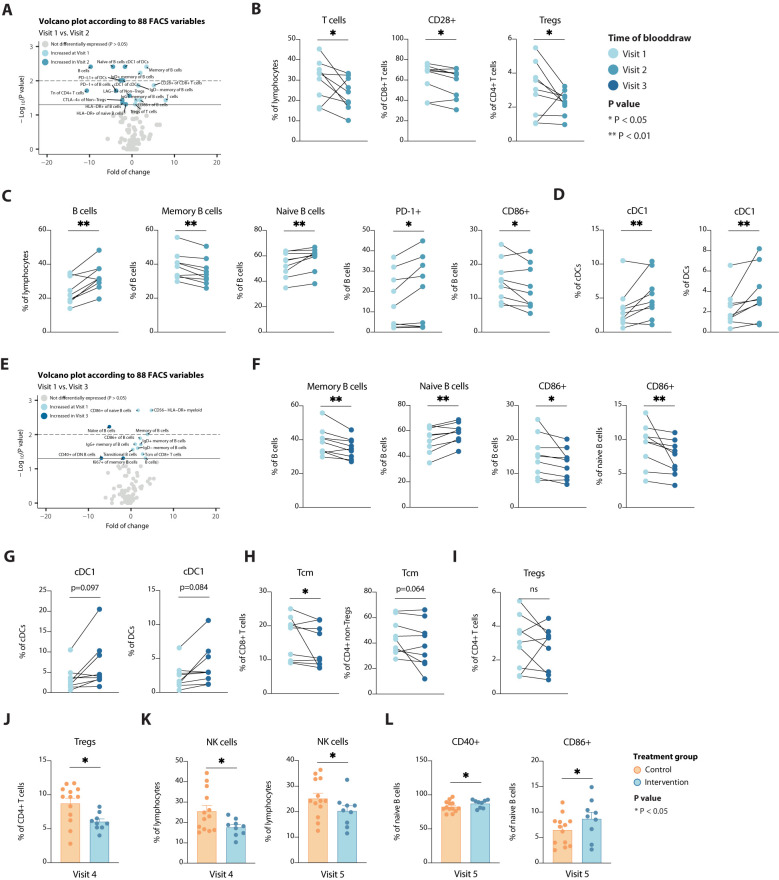
Alterations in immune cell populations over time and according to a STF-diet. **(A)** Volcano plot depicting the fold of change of 88 FACS variables between the Visit 1 and Visit 2 in the intervention group. The x-axis represents the log2 fold change, whereas the y-axis represents -log10 P values. Variables on the right (positive) are increased at Visit 1, and those on the left (negative) are increased at Visit 2. **(B-D)** Line plots showing alterations in T cells **(B)**, B cells **(C)** and dendritic cells **(D)**. **(E)** Volcano plot depicting the fold of change of 88 FACS variables between the Visit 1 and Visit 3 in the intervention group. The x-axis represents the log2 fold change, whereas the y-axis represents -log10 P values. Variables on the right (positive) are increased at Visit 1, and those on the left (negative) are increased at Visit 3. **(F-I)** Line plots showing alterations in B cells **(F)**, dendritic cells **(G)**, T cells **(H)** and regulatory T cells **(I)**. **(J-L)** Bar plots showing differences in regulatory T cells **(J)**, NK cells **(K)** and B cells **(L)** between the two treatment groups at visit 4 or visit 5, as indicated. Tregs, regulatory T cells; cDC, conventional dendritic cells; Tcm, central memory T cells. * = p < 0.05, ** = p <0.01.

In contrast to patients that employed no preoperative diet, STF led to more changes in different immune cell populations, among B cells, T cells and DCs. Preoperatively, circulating T cells were less abundant and less activated, but also immune suppressive regulatory T cells (Tregs) were less abundant after fasting for approximately 40 hours (**Figures 4A, B**). Concomitantly, (naïve) B cells expressing PD-1 and HLA-DR became more abundant in the periphery (**Figure 4C**), suggesting a reduction in specialized B cell subsets and/or B cell development. Finally, cross-presenting type 1 conventional DCs (cDC1s) increased upon caloric restriction (**Figure 4D**), implying the potential to initiate a type 1 cytotoxic immune response, e.g. against viral infection after STF. At time of surgery, solely those changes observed in B cells and cDC1s remained (borderline) significantly different as compared to baseline (**Figures 4E-G**). However, CD8+ and CD4+ Tcm cells were decreased at this stage (**Figure 4H**), suggesting an arrest in T cell development, as seen for B cells (**Figure 4F**). Even though Tregs were only significantly decreased at Visit two, these still trended to be lower at Visit 3 following a STF-diet (**Figure 4I**). Additionally, these reduced levels remained significantly present at time of discharge compared to patients not adhering to a STF-diet (**Figure 4J**). None of the other markers that significantly changed upon STF remained significantly different between the two intervention groups at time of discharge or 6 weeks after surgery (data not shown), indicating that refeeding reshapes the peripheral immune system back to its pre-treatment composition again. Even though STF did not affect the number of circulating NK cells, after surgery control participants did show significantly higher levels than participants adhering to a STF-diet, up to 6 weeks after surgery (**Figure 4K**). In addition, STF led to higher levels of CD86+ and CD40+ naïve B cells 6 weeks after surgery (**Figure 4L**), indicating less specialized cells become activated. Together these changes seen in patients that preoperatively adhere to a STF-diet suggest reduced lymphoid cell activation and development.

## Discussion

In this study, using flow cytometry analysis, we found that fasting changes the abundance and phenotype of peripheral immune cells. Thirty participants were sequentially enrolled in this sub-study. Despite some exclusions due to sample constraints and cancellation of surgery, baseline characteristics were well-matched between the control and intervention groups. Comparable clinical outcomes were observed, and flow cytometry analysis revealed no significant differences between baseline samples from control and intervention groups.

In the control arm of the trial, minimal variables were differentially expressed. Considering that only few variables were DE in this control arm, at admittance or after a limited preoperative fasting duration, we believe these changes are spurious and can be considered not clinically relevant. These results are significant in the context of recent interest in DN B cells, which have been implicated in various diseases, including autoimmune and infectious diseases and cancer (53, 54). However, the effect was deemed minimal, particularly since it vanished at the time of surgery, and it did not show a definite profile with regards to more or less activation. Furthermore, at the time of surgery, when the control group underwent a mean fasting time of 12.2 hours, most variables ceased to be DE. This finding is intriguing given the role of CD86-positive B cells in antigen presentation and immune stimulation (55, 56). Interestingly, the discrepancy between these results and those observed before suggests the likelihood of spurious findings. This underscores the complexity of immune responses and the need for cautious interpretation of experimental results.

In contrast to patients in the control group, STF led to more changes in different immune cell populations, among B cells, T cells and cross-presenting DCs at Visit two. Initially, it was observed that preoperative fasting of approximately 40 hours duration, led to a decrease in circulating T- cells, including Tregs, known for their crucial role in immune regulation and homeostasis (57–59). Even though this finding did not hold significance at time of surgery, patients following an STF-diet still trended toward lower levels of peripheral Tregs. In addition, at time of discharge again these patients showed significant reduced levels of Tregs. These findings are intriguing considering the multifaceted functions of Tregs, which not only suppress immune responses but can also exhibit proinflammatory properties under certain circumstances (40–42). Despite these observations, the implications of these alterations remain unclear, warranting further investigation into the consequences of reduced T cell abundance following prolonged fasting. Additionally, we also found an increase in peripheral (naïve) B cells, suggesting a shift in specialized B-cell subsets or B-cell development.

Finally, an increase in cross-presenting type 1 conventional dendritic cells was observed, known for their pivotal role in orchestrating antitumor immune responses (60, 61). This heightened presence of cDC1s suggests an enhanced potential for initiating type 1 cytotoxic immune responses (60, 61), particularly against viral infections. This finding implicates a potential shift toward a more vigilant immune state against pathogens, of which the clinical significance remains yet unknown. Finally, at the time of surgery with maximum fasting duration, several of these changes persisted, notably in B cells and cDC1s, while CD8+ and CD4+ Tcm cells exhibited a decrease, indicating an arrest in T cell development akin to that observed in B cells. Our data are consistent with previous studies (40–42), showing a decrease in specialized immune cell subsets during a fasting period and rejuvenation of the immune system after refeeding. In addition, these findings highlight the intricate relationship between fasting and immune modulation, warranting further investigation to elucidate the underlying mechanisms and therapeutic implication.

Findings from our study are subject to several limitations. Firstly, the flow cytometry analysis was not performed on all intended participants, as data was missing due to logistical constraints and unexpected changes in clinical treatment. However, we hypothesize that this is non-selective missingness, as it is purely influenced by chance, that aside from limiting the power of our study, induces no significant bias. Secondly, our analyses were limited to peripheral blood. It therefore remains unclear whether the changes seen in blood reflect those in tissues, or whether a decrease of specialized immune cells is due to infiltration within peripheral tissues. Furthermore, as living kidney donors are required to be healthy, we selected by design on a healthy population, thereby limiting generalizability of our findings. The current findings have significant implications for understanding the effects of STF on immune cell populations.

Despite the limitations, the study demonstrates that STF induces distinct changes in immune cell subsets, particularly among B cells, T cells, and DCs. These findings contribute to the growing body of literature on the immunological effects of dietary interventions. Previous research (40–42) namely indicates that STF can modulate immune cell populations. However, the specific changes observed in this study, such as the increase in cDC1s and the reduction in Tregs, provide novel insights into the mechanisms underlying the immunomodulatory effects of fasting. Moving forward, further research is needed to elucidate the effects of STF on immune function and its combined influence on postoperative recovery. Additionally, future studies should aim to address the limitations identified in this study, such as the selection bias due to the study design, to strengthen the generalizability of the findings. Moreover, investigating the potential clinical implications of the observed immunological changes could inform the development of novel therapeutic strategies for immune-related disorders.

## Conclusion

In this study, we found that STF changes the expression of immune cells, reducing abundance and activation of (central memory) T cells and Tregs, increased presence of (naïve) B cells, and elevation of cDC1s. Except for reduced levels of Tregs, none of the markers that significantly changed upon STF remained present at time of discharge, indicating that refeeding reshapes the peripheral immune system back to its pre-fasting composition again. Further research should focus on the clinical implications of the observed immunological changes during STF.

## Data Availability

The datasets generated for this study can be found in the EGA with below detailed EGA-numbers: Title: “Short-term fasting before living kidney donation has an immune-modulatory effect” EGA Study: EGAS00001008034EGA Dataset RNA: EGAD00001015472.
