# Supplementary material for: Short-term fasting before living kidney donation has an immune-modulatory effect
Source: Front Immunol. 2025 Feb 20;16:1488324. doi: 10.3389/fimmu.2025.1488324 (PMC11882433; doi:10.3389/fimmu.2025.1488324)

**Supplementary Tables**

**Supplementary Table 1. Antibodies used for flow cytometry staining.**

| Marker | Fluorochrome | Clone | Staining | Company | Catalog # |
| --- | --- | --- | --- | --- | --- |
| CD3 | AF700 | UCHT1 | Extracellular | eBioscience | 56-0038-42 |
| CD3 | APC-eF780 | UCHT1 | Extracellular | invitrogen | 47-0038-42 |
| CD4 | BV785 | SK3 | Extracellular | BD | 563877 |
| CD8 | BB790-P | SK1 | Extracellular | BD | 624296 |
| CD11c | BV605 | 3.9 | Extracellular | Biolegend | 301635 |
| CD14 | BV785 | M5E2 | Extracellular | BD | 563699 |
| CD15 | APC | HI98 | Extracellular | Biolegend | 301908 |
| CD16 | FITC | 3G8 | Extracellular | BD | 555406 |
| CD19 | AF700 | HIB19 | Extracellular | eBioscience | 56-0199-42 |
| CD19 | BV750 | HIB19 | Extracellular | BD | 747083 |
| CD20 | AF700 | 2H7 | Extracellular | BD | 560631 |
| CD21 | PE-CF594 | B-Ly4 | Extracellular | BD | 563474 |
| CD24 | APC-Cy7 | ML5 | Extracellular | Biolegend | 311132 |
| CD27 | BV421 | M-T272 | Extracellular | BD | 562513 |
| CD28 | PE-Cy7 | CD28.2 | Extracellular | Biolegend | 302926 |
| CD38 | PerCP-Cy5.5/BB700 | HIT2 | Extracellular | BD | 566446 |
| CD39 | BV750 | Tu66 | Extracellular | BD | 747079 |
| CD40 | PE | 5C3 | Extracellular | BD | 555589 |
| CD45RA | PE-TxR | MEM-56 | Extracellular | Invitrogen | MHCD45RA17 |
| CD56 | Pe-Cy7 | B159 | Extracellular | BD | 557747 |
| CD56 | BV605 | NCAM16.2 | Extracellular | BD | 562780 |
| CD86 | Biotin | 2331 (FUN1) | Extracellular | BD | 555656 |
| CD86 | BV650 | 2331 (FUN1) | Extracellular | BD | 563411 |
| CD123 | BV650 | 7G3 | Extracellular | BD | 563405 |
| CD137L | BV421 | C65-485 | Extracellular | BD | 744392 |
| CD152 (CTLA-4) | PerCP-ef710 | 14D3 | Extracellular | eBioscience | 46-1529-42 |
| CD197 (CCR7) | BV421 | G043H7 | Extracellular | Biolegend | 353208 |
| CD223 (LAG-3) | AF700 | 11C3C65 | Extracellular | Biolegend | 369343 |
| CD278 (ICOS) | BV650 | DX29 | Extracellular | BD | 563832 |
| FoxP3 | PE | 236A/E7 | Intracellular | eBioscience | 12-4777-42 |
| HLA-DR | BV711 | G46-6 | Extracellular | BD | 563696 |
| IgD | PE-Cy7 | IA6-2 | Extracellular | BD | 561314 |
| IgG | BV786 | G-18-145 | Extracellular | BD | 564230 |
| IgM | BV605 | G20-127 | Extracellular | BD | 562977 |
| IRF4 | PE | 3E4 | Intracellular | eBioscience | 12-9858-82 |
| IRF8 | PerCp-Cy5.5/BB700 | V3GYWCH | Intracellular | eBioscience | 45-9852-82 |
| Ki67 | FITC | 20Raj1 | Intracellular | Invitrogen | 11-5699-42 |
| PD-1 | APC | EH12.2H7 | Extracellular | Biolegend | 329907 |
| PD-L1 | PE-CF594 | M1H1 | Extracellular | BD | 563742 |
| Streptavidin | APC-Cy7 | n/a | Extracellular | Invitrogen | 47-4317-82 |
| Aqua Live/Dead | eFluor 506 | n/a | n/a | eBioscience | 65-0866-14 |
| Human TruStain | n/a | n/a | n/a | Biolegend | 422302 |

**Supplementary Table 2. Flow cytometry data output.**

| **FACS variable** |
| --- |
| % Myeloid cells of live cells |
| % HLA-DR+ of myeloid cells |
| % CD56- of HLA-DR+ myeloid cells |
| % Monocytes of HLA-DR+ CD56- myeloid cells |
| % CD86+ of monocytes |
| % CD137L+ of monocytes |
| % PD-L1+ of monocytes |
| % Classical monocytes of monocytes |
| % Non-classical monocytes of monocytes |
| % Intermediate monocytes of monocytes |
| % Classical monocytes of myeloid cells |
| % Non-classical monocytes of myeloid cells |
| % Intermediate monocytes of myeloid cells |
| % DCs of myeloid cells |
| % CD86+ of DCs |
| % CD137L+ of DCs |
| % PD-L1+ of DCs |
| % CD11c low of DCs |
| % pDC of DCs |
| % cDC of DCs |
| % cDC1 of cDCs |
| % cDC2 of cDCs |
| % cDC1 of DCs |
| % cDC2 of DCs |
| % B cells of lymphocytes |
| % Non-transitional, non-plasmablasts of B cells |
| % Plasmablasts of B cells |
| % Transitional B cells of B cells |
| % Memory of B cells |
| % Naïve of B cells |
| % DN of B cells |
| % IgD+ memory of B cells |
| % IgD- memory of B cells |
| % IgG+ memory of B cells |
| % IgM+ memory of B cells |
| % CD40+ of B cells |
| % CD86+ of B cells |
| % HLA-DR+ of B cells |
| % Ki67+ of B cells |
| % PD-1+ of B cells |
| % CD40+ of memory B cells |
| % CD86+ of memory B cells |
| % HLA-DR+ of memory B cells |
| % Ki67+ of memory B cells |
| % PD-1+ of memory B cells |
| % CD40+ of naive B cells |
| % CD86+ of naive B cells |
| % HLA-DR+ of naive B cells |
| % Ki67+ of naive B cells |
| % PD-1+ of naive B cells |
| % CD21+ of DN B cells |
| % CD21- of DN B cells |
| % CD40+ of DN B cells |
| % CD86+ of DN B cells |
| % HLA-DR+ of DN B cells |
| % Ki67+ of DN B cells |
| % PD-1+ of DN B cells |
| % NK cells of lymphocytes |
| % NKT cells of lymphocytes |
| % T cells of lymphocytes |
| % CD4+ of T cells |
| % Tregs of CD4+ T cells |
| % Tregs of T cells |
| % Non-Tregs of CD4+ T cells |
| % Tcm of CD4+ non-Tregs |
| % Tem of CD4+ non-Tregs |
| % Temra of CD4+ non-Tregs |
| % Tn of CD4+ non-Tregs |
| % CD28+ of Non-Tregs |
| % CTLA-4+ of Non-Tregs |
| % HLA-DR+ of Non-Tregs |
| % ICOS+ of Non-Tregs |
| % Ki67+ of Non-Tregs |
| % LAG-3+ of Non-Tregs |
| % PD-1+ of Non-Tregs |
| % CD8+ of T cells |
| % Tcm of CD8+ T cells |
| % Tem of CD8+ T cells |
| % Temra of CD8+ T cells |
| % Tn of CD8+ T cells |
| % CD28+ of CD8+ T cells |
| % CD39+ of CD8+ T cells |
| % CTLA-4+ of CD8+ T cells |
| % HLA-DR+ of CD8+ T cells |
| % ICOS+ of CD8+ T cells |
| % Ki67+ of CD8+ T cells |
| % LAG-3+ of CD8+ T cells |
| % PD-1+ of CD8+ T cells |

**Supplementary Figures**

***Supplementary Figure 1. Gating strategy for individual cell subsets.*** *Abbreviations: NK, natural killer cells; NKT, natural killer T cells; T, T cells; Tregs, regulatory T cells; Temra, terminally differentiated effector T cells; Tn, naïve T cells; Tcm, central memory T cells; Tem, effector memory T cells; DN, double negative; PB, plasmablasts; Trans, transitional B cells; cDC, conventional dendritic cells; pDC, plasmacytoid dendritic cells.*

***
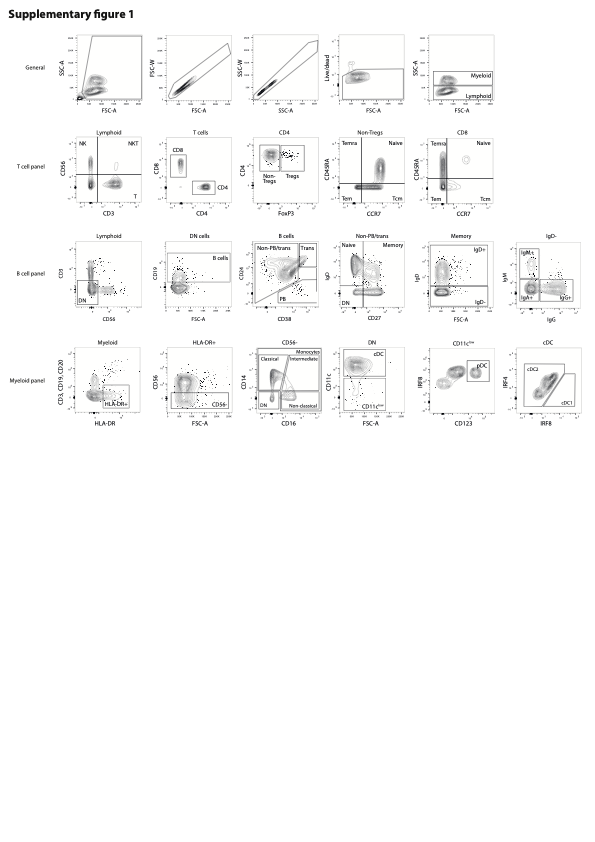
***

***Supplementary Figure 2. Difference in clinical parameters between study groups.*** *(A-F) Boxplots showing differences in weight (A), weightloss during fasting period (B) or waiting time (C), glucose (D), insulin (E) and BHB-value (F) at admission between study groups.*


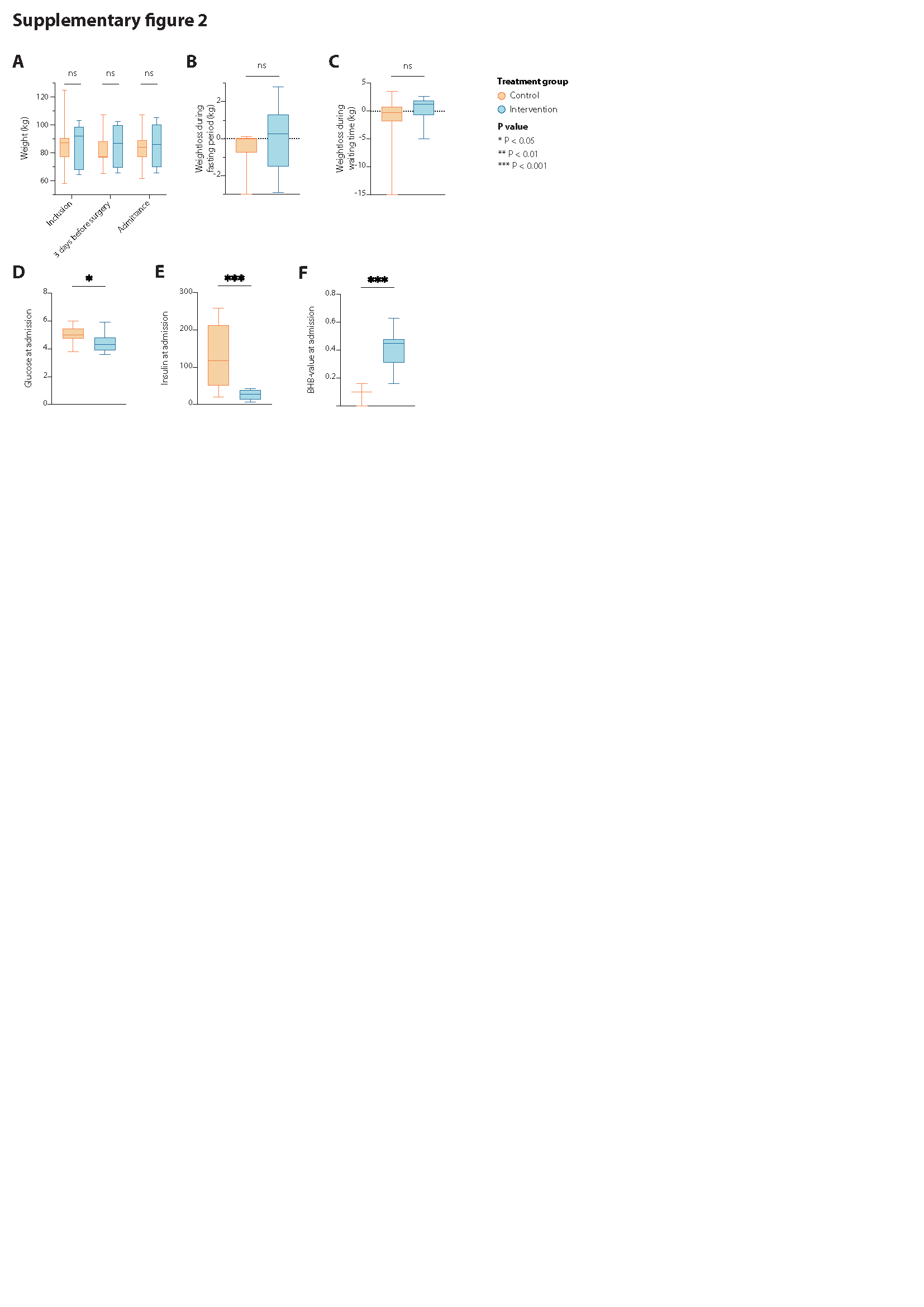
**Supplementary Figure 3. *Gene-set enrichment analysis (GSEA) between treatment groups.*** Gene-set enrichment analysis (GSEA) between treatment groups. **(A-B)** Bubble plot summary of GSEA top significant Immune Related Reactome Pathways **(A)** and Cell Type Signature Gene Sets **(B)**. Red dots have a positive normalized enrichment score (NES), meaning upregulated, and blue dots negative NES, thus downregulated. Size of dots indicates size of corresponding gene set.


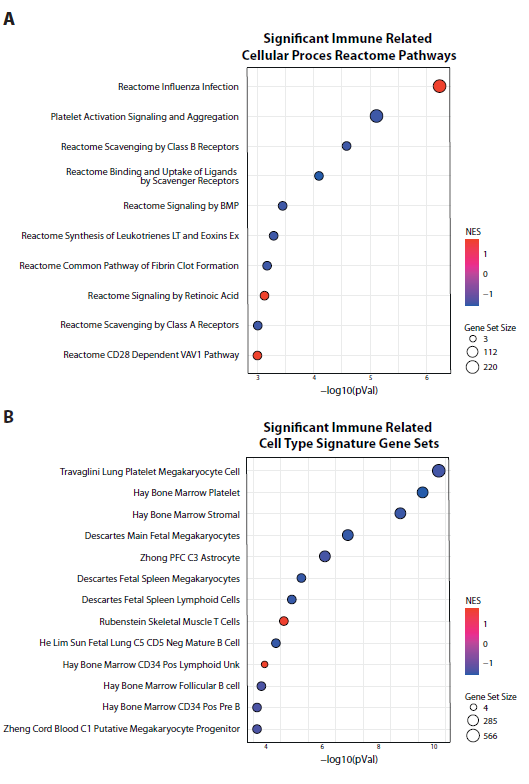

Supplement: Supplementary file 2 [file DataSheet1.docx]
